# Growth of *Chlamydomonas reinhardtii* in acetate-free medium when co-cultured with alginate-encapsulated, acetate-producing strains of *Synechococcus* sp. PCC 7002

**DOI:** 10.1186/s13068-014-0154-2

**Published:** 2014-10-18

**Authors:** Jesse B Therien, Oleg A Zadvornyy, Matthew C Posewitz, Donald A Bryant, John W Peters

**Affiliations:** Department of Chemistry and Biochemistry, Montana State University, Bozeman, Montana 59717 USA; Department of Biochemistry and Molecular Biology, The Pennsylvania State University, University Park, Pennsylvania 16802 USA; Department of Chemistry and Geochemistry, Colorado School of Mines, Golden, Colorado 80401 USA

**Keywords:** Biofuels, Algae, Cyanobacteria, Lipid production, Acetate production, Co-culture, Alginate immobilization

## Abstract

**Background:**

The model alga *Chlamydomonas reinhardtii* requires acetate as a co-substrate for optimal production of lipids, and the addition of acetate to culture media has practical and economic implications for algal biofuel production. Here we demonstrate the growth of *C. reinhardtii* on acetate provided by mutant strains of the cyanobacterium *Synechococcus* sp. PCC 7002.

**Results:**

Optimal growth conditions for co-cultivation of *C. reinhardtii* with wild-type and mutant strains of *Synechococcus* sp. 7002 were established. In co-culture, acetate produced by a glycogen synthase knockout mutant of *Synechococcus* sp. PCC 7002 was able to support the growth of a lipid-accumulating mutant strain of *C. reinhardtii* defective in starch production. Encapsulation of *Synechococcus* sp. PCC 7002 using an alginate matrix was successfully employed in co-cultures to limit growth and maintain the stability.

**Conclusions:**

The ability of immobilized strains of the cyanobacterium *Synechococcus* sp. PCC 7002 to produce acetate at a level adequate to support the growth of lipid-accumulating strains of *C. reinhartdii* offers a potentially practical, photosynthetic alternative to providing exogenous acetate into growth media.

**Electronic supplementary material:**

The online version of this article (doi:10.1186/s13068-014-0154-2) contains supplementary material, which is available to authorized users.

## Background

With the ongoing increase in global energy demand, the development of alternative energy sources has been at the forefront of recent modern research. Of the various alternative energies, bioenergy has been of interest because of its many potential benefits over the currently used petroleum, natural gas, and coal. These benefits include carbon neutrality, renewability, low environmental toxicity, and reduction of dependence on foreign energy sources. Biodiesel and bioethanol, which together currently account for the major proportion of biofuels, are mainly produced from higher plants [1]. These fuels can replace diesel and gasoline, respectively, in conventional engines without modification. Of these two biofuels, biodiesel has been considered the most viable option in the United States [2]. However, the production of biodiesel from crops grown on arable land is controversial due to the potential implications of the competition with agricultural food production [3,4]. One alternative to using food crops as oil sources is to produce biofuels using microalgae and/or cyanobacteria, which can be grown in areas not suitable for crop growth, such as deserts [5,6]. Microalgae can be used to assimilate CO_2_ and produce multiple fuel molecules, including hydrogen, starch, and lipids [7–9]. Starch can be fermented to hydrogen or ethanol, while lipids can be converted to biodiesel [10–12].

Some lipid-accumulating microalgae, such as *Chlamydomonas reinhardtii*, are able to grow photoautotrophically on sunlight and CO_2_, chemotrophically on acetate, or photomixotrophically in a combination of these two growth modes [13,14]. Optimal lipid production by *C. reinhardtii* is observed under photomixotrophic conditions in the presence of acetate, which could present economic and practical challenges for large-scale production of algal-based biofuels [15,16]. An alternative is to co-culture lipid-producing *C. reinhardtii* with an acetate-producing cyanobacterium, such as certain *Synechococcus* sp., that can naturally produce acetate during photosynthesis and/or fermentation [17–19].

In this study, we present results of co-culturing wild-type and a lipid-accumulating (*sta6*) strain of *C. reinhardtii*, and an acetate-accumulating strain (*glgA1*) of *Synechococcus* sp. PCC 7002. We demonstrate sustained photomixotrophic growth of *C. reinhardtii* with acetate produced by *Synechococcus* 7002.

## Results and discussion

### Effect of temperature on growth of *Synechococcus* sp. PCC 7002 and *C. reinhardtii*

**Figure 1:**
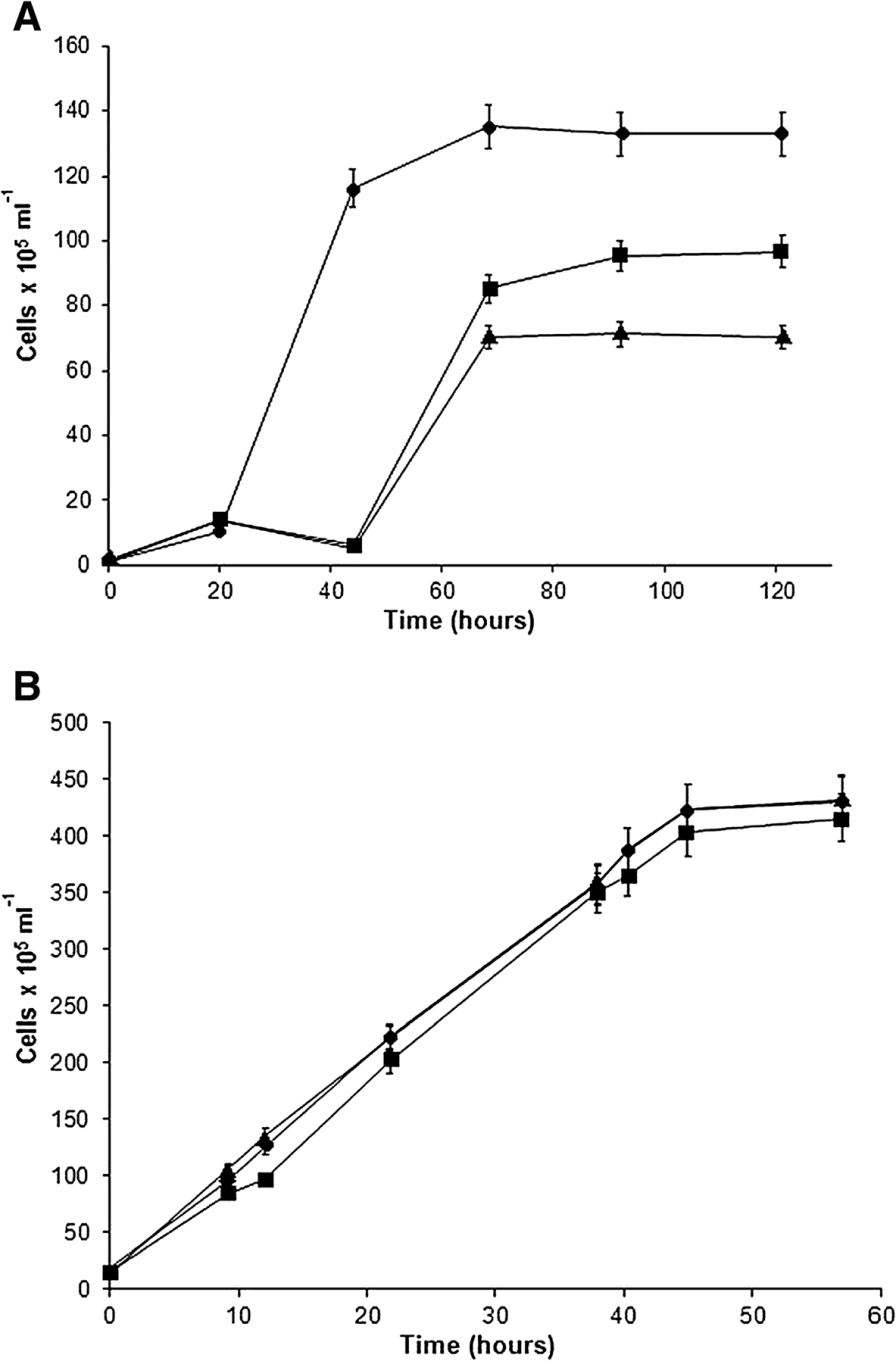
**Effect of temperature on growth of** ***C. reinhartdii*** **and** ***Synechococcus*** **sp** ***.*** **PCC 7002.** Cultures of *C. reinhartdii* **(A)** and *Synechococcus* sp*.* PCC 7002 **(B)** were grown at 30°C (circles), 34°C (squares), and 38°C (triangles).

To determine conditions under which both organisms could grow well together, the wild-type and mutant strains of *C. reinhardtii* and *Synechococcus* sp. PCC 7002 were grown individually on Tris-Acetate-Phosphate (TAP) medium, a standard medium for *C. reinhardtii*, and A^+^ medium, a standard medium for *Synechococcus* sp. PCC 7002. Consistent with previous reports, the results of our preliminary experiments showed that the optimum temperature for the growth of *C. reinhardtii* is ~30°C [20–22] and that for *Synechococcus* 7002 ~ 38°C [23]. An increase in temperature from 30°C to 34°C and then to 38°C increased the lag phase in the *C. reinhardtii* cultures (Figure 1A). In contrast, decreasing the temperature from 38°C to 34°C and finally to 30°C did not significantly affect the growth of either wild-type (Figure 1B) or mutant strains of *Synechococcus* sp. PCC 7002 under the light intensities and CO_2_ concentrations used in this study (data not shown). Therefore subsequent co-culture optimizations were conducted at 30°C.

### Effect of media composition on growth of *C. reinhardtii* and *Synechococcus* sp. PCC 7002

**Figure 2:**
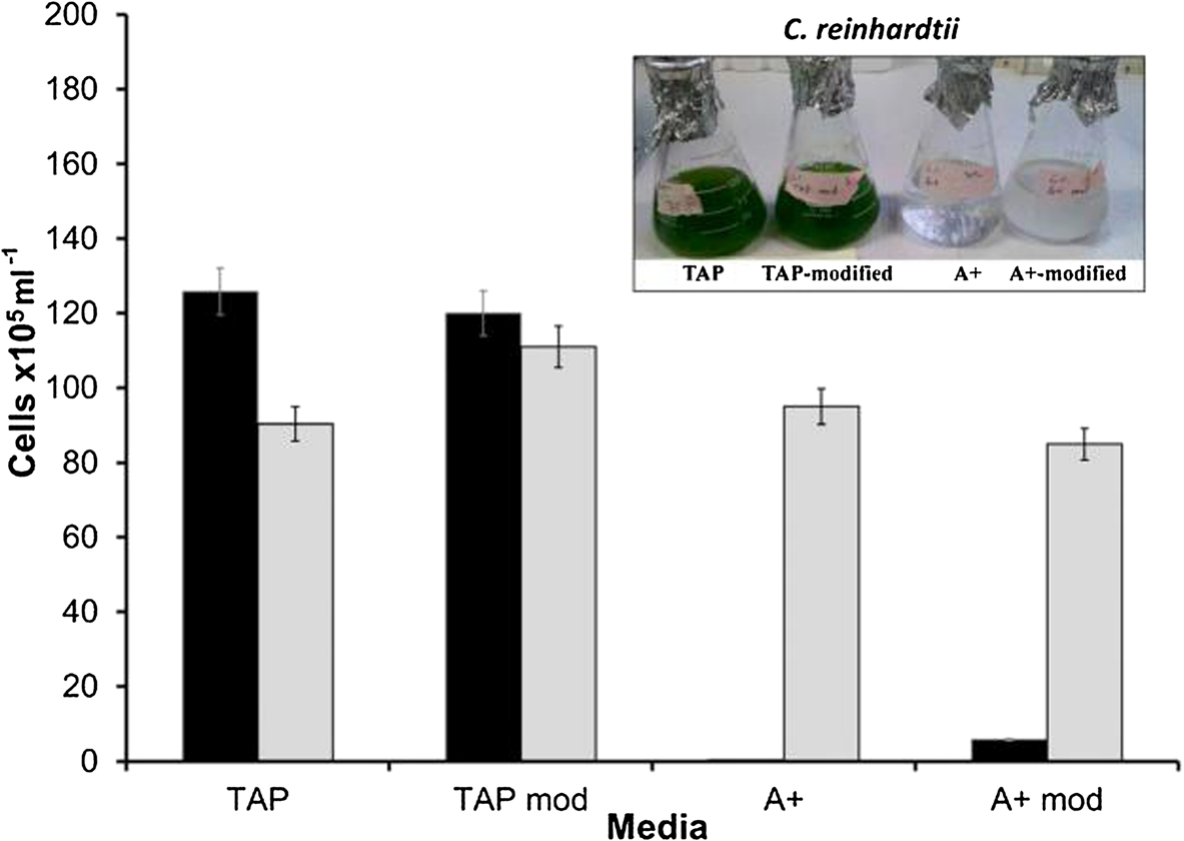
**Growth of** ***C. reinhartdii*** **and** ***Synechococcus*** **sp. PCC 7002 on different types of media.** Cultures of *C. reinhartdii* (black bars) and *Synechococcus* sp*.* PCC 7002 (gray bars) were grown on TAP, co-culture media (TAP mod), A^+^, modified A^+^ media at 30°C after 124 hours. The difference in the media composition is described in the text and in the Methods section.

The main difference between TAP medium (*C. reinhardtii*) and A^+^ medium (*Synechococcus* sp. PCC 7002) is that medium A^+^ is a marine medium that contains 300 mM sodium chloride. Other differences include the use of ammonium chloride as the nitrogen source in TAP medium versus sodium nitrate in A^+^ medium, the presence of acetate in TAP, and the presence of vitamin B_12_ in A^+^ medium. To accommodate the growth of both *C. reinhardtii* and *Synechococcus* sp. PCC 7002 in co-culture, both the TAP and A^+^ media were modified (henceforth referred to as modified TAP and modified A^+^, respectively). Modified TAP medium consists of TAP medium supplemented with sodium nitrate and vitamin B_12_; modified A^+^ medium consists of standard A^+^ medium supplemented with ammonium chloride and acetic acid, with the sodium chloride concentration reduced to 150 mM. (Additional file 1: Table S1). The results of our experiments showed that *C. reinhardtii* cultures are unable to grow on A^+^ medium and/or modified A^+^ medium at 30°C (Figure 2, and insert in Figure 2) and 38°C (data not shown). However, *Synechococcus* sp. PCC 7002 was able to grow on both TAP and modified TAP media at 30°C (Figure 2).

### Effect of acetate on growth of *C. reinhardtii* and *Synechococcus* sp. PCC 7002

**Figure 3:**
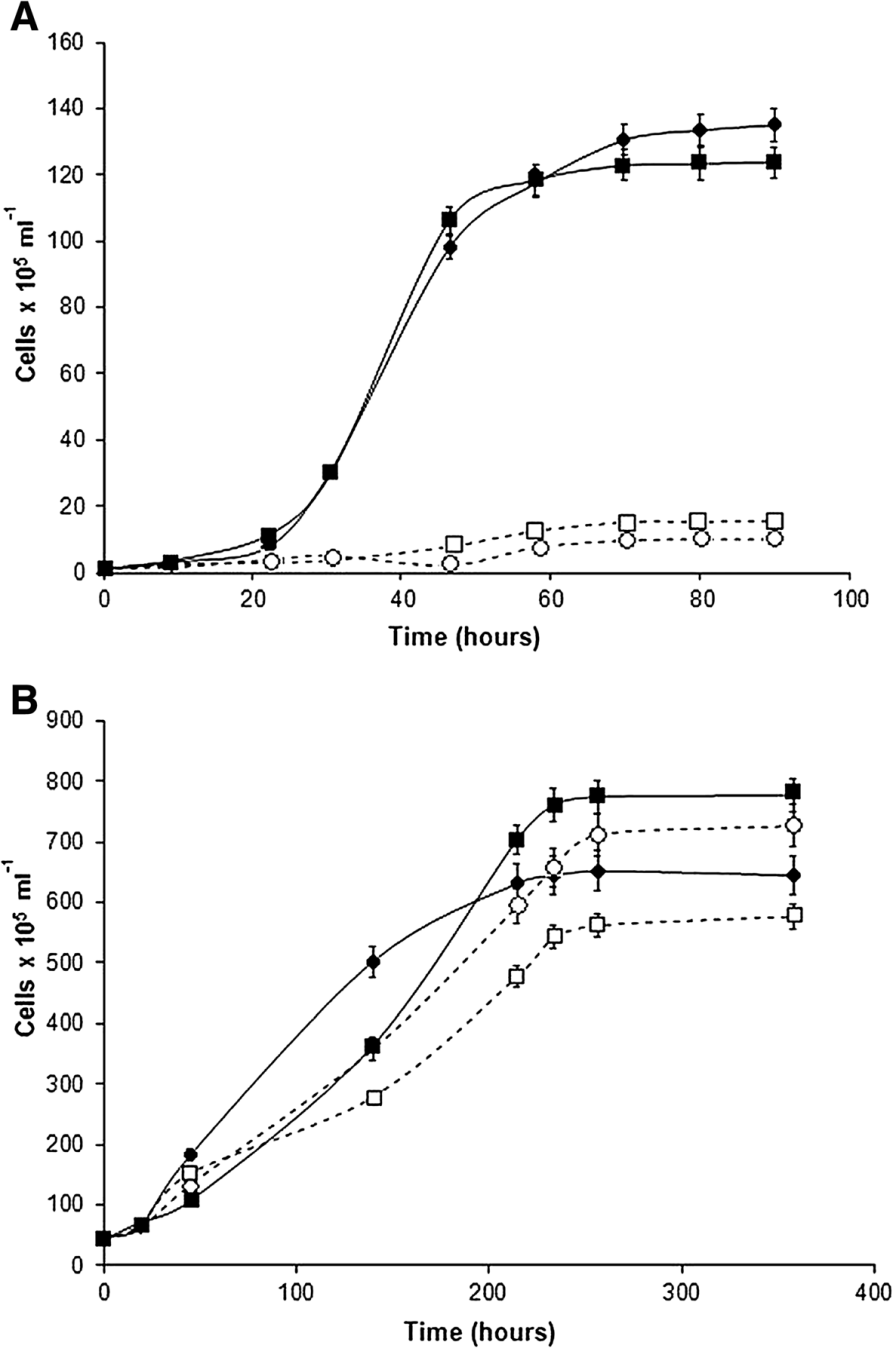
**Effect of acetate on growth of** ***C. reinhartdii*** **and** ***Synechococcus*** **sp. PCC 7002.** The cultures of *C. reinhartdii* **(A)** and *Synechococcus* sp*.* PCC 7002 **(B)** were grown in the presence (closed symbols) and absence (open symbols) of acetate. Both *C. reinhartdii* and *Synechococcus* sp*.* PCC 7002 wild-type cultures are shown in circles. The *sta6* mutant of *C. reinhartdii* and the *glgA* mutant of *Synechococcus* sp*.* PCC 7002 are shown in squares.

**Figure 4:**
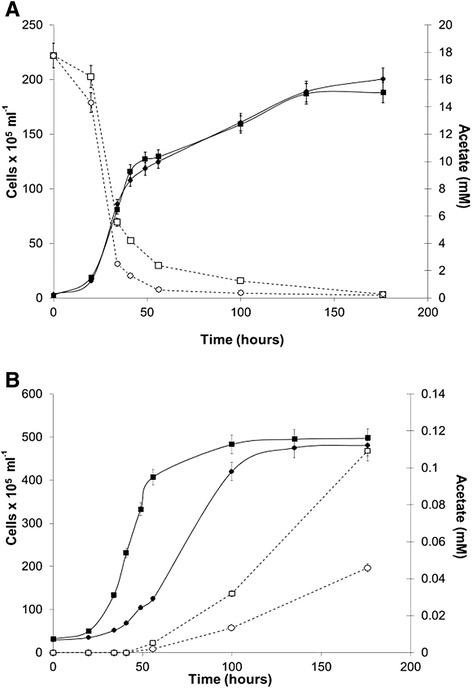
**Growth and consumption of acetate by** ***C. reinhartdii*** **cultures, and growth and production of acetate by** ***Synechococcus*** **sp. PCC 7002 cultures.** The cultures of wild-type (closed circles) and *sta6* mutant (closed squares) of *C. reinhartdii* **(A)** and wild-type (closed circles) and *glgA* (closed squares) of *Synechococcus* sp*.* PCC 7002 **(B)** grown on co-culture media at pH 7.0 and 30°C. Acetate consumption **(A)** by wild-type (open circles) and *sta6* mutant (open squares) *C. reinhartdii* and production **(B)** by wild-type (open circles) and *glgA* mutant (open squares) *Synechococcus* sp*.* PCC 7002 were measured during the cell growth.

To investigate the effects of acetate on the growth of both wild-type and mutant cultures of *C. reinhardtii* and *Synechococcus* sp. PCC 7002, cultures were grown on TAP medium containing acetate and on a modified form of TAP medium that lacked acetate (termed co-culture medium). The results of these experiments showed that the growth rates of wild-type and mutant cultures of *C. reinhartdii* were higher in the presence of acetate when compared to cultures grown in the absence of acetate. Cultures of *C. reinhardtii* grown in acetate-free medium showed little growth under these conditions (Figure 3A). Although *Synechococcus* sp. PCC 7002 cultures grown without acetate had slightly lower final cell densities than cultures grown in the presence of acetate, the growth rates with and without acetate were similar for both wild-type and mutant strains (Figure 3B). During the growth of wild-type and *sta6* mutant strains of *C. reinhardtii,* acetate consumption correlated with an increase in cell number (Figure 4A). Acetate production by the *glgA1* mutant of *Synechococcus* 7002 was higher than that of the wild type (Figure 4B).

### Optimizing co-cultures

**Figure 5:**
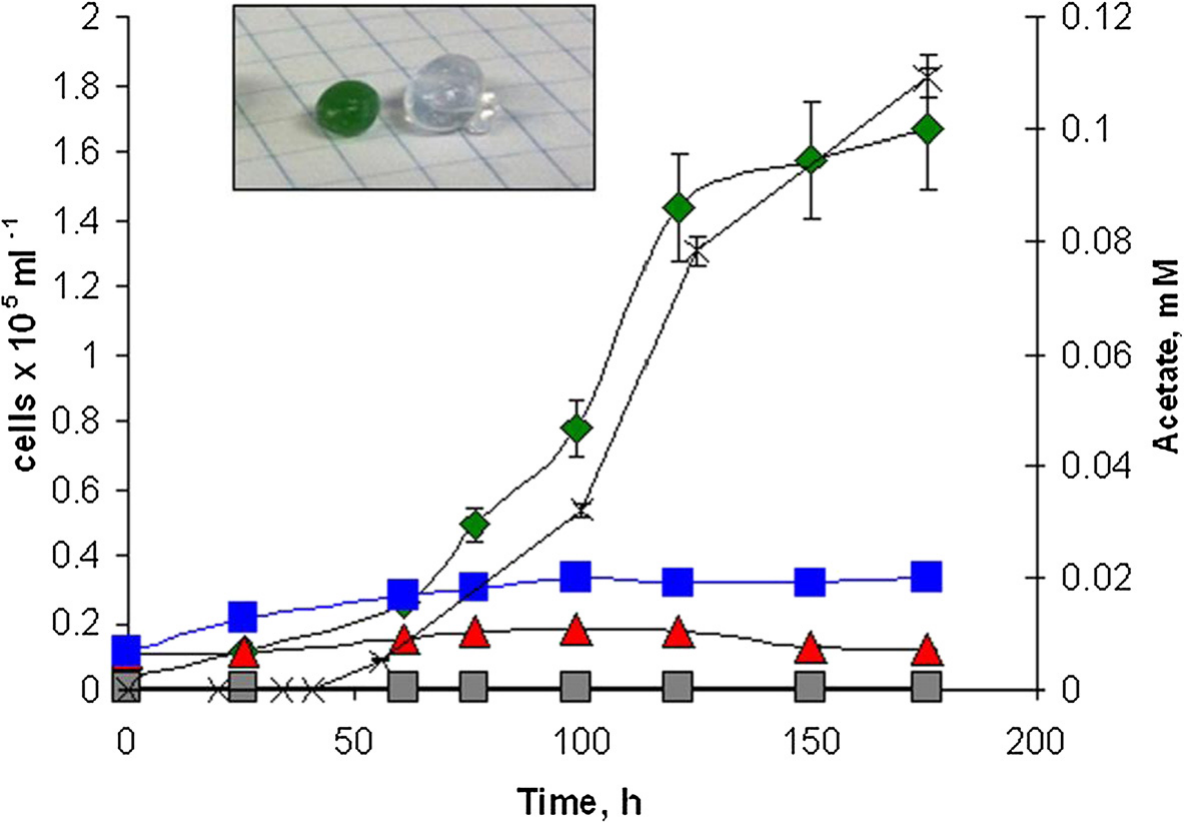
**Co-culturing** ***C. reinhartdii*** **and** ***Synechococcus*** **sp. PCC 7002.** The *C. reinhartdii sta6* mutant was grown with alginate bead immobilized *Synechococcus* sp. PCC 7002 wild-type cells (blue squares), *glgA1* cells (green diamonds), and with empty beads (red triangles) in co-culture on modified TAP media without acetate at pH 7.0. Growth of *Synechococcus* sp. PCC 7002 cells leaking out of the beads is shown in gray squares. Acetate production by alginate bead immobilized *Synechococcus* sp. PCC 7002 *glgA1* mono-culture is shown in stars. The inset shows alginate beads of *Synechococcus* sp. (left) and an empty bead (right).

Based on the initial experiments, the *sta6* mutant of *C. reinhardtii* was grown on co-culture medium at pH 7.0 and 30°C with the *glgA1* mutant of *Synechococcus* sp. PCC 7002. To investigate the effect of initial cell number in the inoculum during co-culturing of free cells, different initial ratios, 1:1 and 1:10, of *C. reinhardtii* to *Synechococcus* sp. PCC 7002 cells were tested. The ratio 1:10 was chosen because *Synechococcus* sp. PCC 7002 appeared to produce less acetate than required to produce optimal growth of *C. reinhardtii*, and it was rationalized that a larger number of *Synechococcus* sp. PCC 7002 cells might be advantageous in the co-cultures. However, the results of the co-culturing experiments with free cells revealed that *Synechococcus* sp. PCC 7002 cells grew faster than *C. reinhardtii* at both initial cell ratios and eventually took over the co-culture (data not shown). To avoid the problem of overgrowth by *Synechococcus* sp. PCC 7002, the cells were encapsulated in alginate beads to slow their growth (see Methods section for details). The growth of the *C. reinhardtii sta6* mutant in the presence of control (empty) beads in modified TAP media showed that the alginate encapsulating matrix had no effect on the growth of *C. reinhardtii* cells (data not shown). The results of the experiments using alginate-encapsulated *Synechococcus* sp. PCC 7002 cells showed that the growth of the lipid-accumulating *C. reinhardtii sta6* strain could be supported by the presence of the acetate-producing *Synechococcus* sp. PCC 7002 *glgA1* strain (Figure 5) and that immobilization controlled the growth of *Synechococcus* sp. PCC 7002 and kept the co-cultures from being overgrown.

## Conclusions

We have developed media formulations and growth conditions that support co-cultures of wild-type and the acetate-producing *glgA1* strain of the *Synechococcus* sp. PCC 7002 and wild-type and lipid-accumulating *sta6* mutant of *C. reinhardtii* (Additional file 2: Figure S1). A temperature of 30°C supported the growth of both organisms; however, *Synechococcus* sp. PCC 7002 overtook the culture when both organisms were present as free cells. Alginate encapsulation was found to be an effective way to slow the growth of *Synechococcus* sp. PCC 7002 while at the same time allowing the production of sufficient acetate to stimulate growth and lipid storage of *C. reinhardtii*. The encapsulation of *Synechococcus* sp. PCC 7002 also provides a potential mechanism for separating and harvesting *C. reinhardtii* for lipids and recycling of the *Synechococcus* sp. PCC 7002 cells. The results reported here provide a strong proof of concept for supplanting the provision of costly medium components through co-culturing that can be optimized through metabolic engineering and implementation of enhanced acetate-producing cyanobacteria.

## Methods

### Growth of microorganisms

Inoculum cultures of both *Synechococcus* sp. PCC 7002 and *C. reinhardtii* (25 ml) were prepared in standard growth media for each organism in 50-ml Erlenmeyer flasks oscillating at 100 rpm at a constant illumination of 100 μmol photons m^-2^ s^-1^ with warm white fluorescent light. Wild-type and *glgA1* knockout mutant strains of *Synechococcus* sp. PCC 7002 were grown in medium A^+^ (pH = 8.0) [24] (Additional file 1: Table S1) and were incubated at 38°C while sparging with 1% v/v CO_2_ in air. Wild-type *C. reinhardtii* and *sta6* mutant strains were grown in TAP media (pH = 7.0) incubated at 30°C. The optical density was monitored at 750 nm (ThermoSpectronic Bio Mate 3 spectrophotometer) for *Synechococcus* sp. PCC 7002 and *C. reinhardtii* strains. Cultures were harvested by centrifugation for 10 min at 8,000 × *g*, washed in the medium to be used, centrifuged again, and finally resuspended in the appropriate medium and used to inoculate different culture conditions for co-culturing. Optimal co-culturing conditions were determined by first growing wild-type *Synechococcus* sp. PCC 7002 and *C. reinhardtii* strains individually at 30°C, 34°C, and 38°C on TAP medium (standard *C. reinhardtii* growth medium) or A^+^ medium (standard *Synechococcus* sp. PCC 7002 growth medium). The formulation of the TAP media, A^+^ medium, and modified derivatives of each can be found in the supplemental data (Additional file 1: Table S1). For the study, standard TAP medium was modified by eliminating acetic acid and supplementing with 1 g NaNO_3_ L^-1^ and 4 μg vitamin B_12_ L^-1^, and this formulation was termed co-culture medium. A^+^ medium was supplemented with 7.5 g NH_4_Cl L^-1^ and 1 ml glacial acetic acid L^-1^, and the concentration of NaNO_3_ and NaCl was reduced by 50%. Co-cultures were inoculated with a mixture of seed cultures grown in the appropriate culture media, and the optical densities were monitored at both 600 nm and 750 nm. The cultures were monitored for bacterial contamination by microscopic examination, and cell numbers were determined by direct cell counting with a hemocytometer.

### Cell immobilization

*Synechococcus* sp. PCC 7002 cultures were harvested at an OD_650 nm_ ranging from 0.8-1.0 (ThermoSpectronic Bio Mate 3 spectrophotometer) via centrifugation and resuspended in 1/5 the original media volume, yielding a concentrated cell suspension. Alginate beads were made by dissolving 3 g of sodium alginate directly in 100 ml of concentrated culture media and adding it dropwise with a syringe and needle into a 1% (w/v) solution of CaCl_2_ in growth media from a height of at least 30 cm [25]. The bead size was controlled by needle gauge: 22 G yields beads approximately 2 mm in diameter and 18 G yields beads of 3 mm, while 4-mm beads are made directly from a 10-ml syringe without a needle. Beads were formed immediately and were allowed to harden further in the CaCl_2_ solution for 15 min. After hardening, the beads were removed from the CaCl_2_ solution and added to fresh growth media. To prevent leakage of cells, a cell-free layer of alginate was used to coat the beads. To produce the coating, the beads were submerged in a 3% (w/v) alginate solution in growth medium followed by transfer to a 1% (w/v) solution of CaCl_2_ in growth medium to allow hardening of the cell-free alginate layer.

### Acetate determination

Acetate concentrations were determined by ^1^H-NMR [26]. Samples were centrifuged for 10 min at 14,000 × *g* and 500 μl of the supernatant was transferred to an NMR tube to which 50 μl of D_2_O was added. Spectra were collected on a Bruker DRX500 NMR spectrometer operating at a frequency of 500.13 MHz with a 1D NOESY pulse sequence and a 100-ms mixing time at 32 K. Typically, 64 4.28-s scans were collected for each sample with a pre-saturation pulse to suppress the water signal.

## Additional files

Additional file 1: Table S1. Media composition of TAP, A^+^, modified TAP, modified A^+^ per liter of dH_2_O. Additional file 2: Figure S1. Growth and lipid accumulation of wild-type and *sta6* mutant *C. reinhardtii*. Cultures of wild-type (closed squares, dotted line) and *sta6* mutant (closed diamonds, dotted line) *C. reinhardtii* were grown on modified TAP media at 30°C in the presence of acetate. The lipid accumulation in wild-type (open squares, solid line) and *sta6* mutant (open diamonds, solid line) *C. reinhardtii* cells was determined with Nile red stain after a 10-min incubation [1]. The amount of lipids accumulated after 42 hours growth of wild-type *C. reinhardtii* cells was chosen as 100%.
