# Supplementary material for: Growth of Chlamydomonas reinhardtii in acetate-free medium when co-cultured with alginate-encapsulated, acetate-producing strains of Synechococcus sp. PCC 7002
Source: Biotechnol Biofuels. 2014 Oct 18;7:154. doi: 10.1186/s13068-014-0154-2 (PMC4216383; doi:10.1186/s13068-014-0154-2)
Supplement: Additional file 1: Table S1. — Media composition of TAP, A+, modified TAP, modified A+ per liter of dH2O. [file 13068_2014_154_MOESM1_ESM.doc]

# Additional files

**Additional file 1: Table S1. Media composition of TAP, A^+^, modified TAP, modified A^+^ per litter of dH_2_O.**

|  | **TAP** | **A**^+^ | **Modified TAP** | **Modified A**^+^ |
| --- | --- | --- | --- | --- |
| TRIS Base | 2.4 g | 1.0 g | 2.4 g | 1.0 g |
| NaNO_3_ | - | 1.0 g | 1.0 g | 0.5 g |
| NH_4_Cl | 15.0 g | - | 15.0 g | 7.5 g |
| MgSO_4_(7H_2_O) | 4.0 g | 5.0 g | 4.0 g | 5.0 g |
| CaCl_2_(2H_2_O) | 2.0 g | 2.7 g | 2.0 g | 2.7 g |
| NaCl | - | 18.0 g | - | 9.0 g |
| KCl | - | 0.6 g | - | 0.6 g |
| K_2_HPO_4_ | 0.3 g | - | 0.3 g | - |
| KH_2_PO_4_ | 0.14 g | 0.05 g | 0.14 g | 0.05 g |
| Na_2_EDTA(2H_2_O) | 50 mg | 30 mg | 50 mg | 30 mg |
| ^(1)^ZnSO_4_/^(2)^ZnCl_2_ | ^(1)^22 mg | ^(2)^0.3 mg | ^(1)^22 mg | ^(2)^0.3 mg |
| H_3_BO_3_ | 11.4 mg | 34.3 mg | 11.4 mg | 34.3 mg |
| MnCl_2_ | 5.1 mg | 4.3 mg | 5.1 mg | 4.3 mg |
| ^(1)^FeSO_4_/^(2)^FeCl_3_ | ^(1)^5.0 mg | ^(2)^3.9 mg | ^(1)^5.0 mg | ^(2)^3.9 mg |
| CoCl_2_ | 1.6 mg | 1.2 mg | 1.6 mg | 1.2 mg |
| CuSO_4_ | 1.6 mg | 3.0 µg | 1.6 mg | 3.0 µg |
| MoO_3_ | 1.10 mg | 0.03 mg | 1.10 mg | 0.03 mg |
| Glacial Acetic Acid | 1 ml | - | 1 ml | 1 ml |
| Vitamin B_12_ | - | 4 µg | 4 µg | 4 µg |
| pH | 7 | 8 | 7 and 8 | 7 and 8 |

**Figure S1. Growth and lipid accumulation of wild type and *sta6* mutant *C. reinhardtii*.** Cultures of wild type (closed squares, dotted line) and *sta6* mutant (closed diamonds, dotted line) *C. reinhardtii* were grown on modified TAP media at 30 °C in the presence of acetate. The lipid accumulation in wild type (open squares, solid line) and *sta6* mutant (open diamonds, solid line) *C. reinhardtii* cells was determined with Nile Red stain after a 10 min incubation [1]. The amount of lipids accumulated after 42 hours growth of wild type *C. reinhardtii* cells was chosen as 100%.


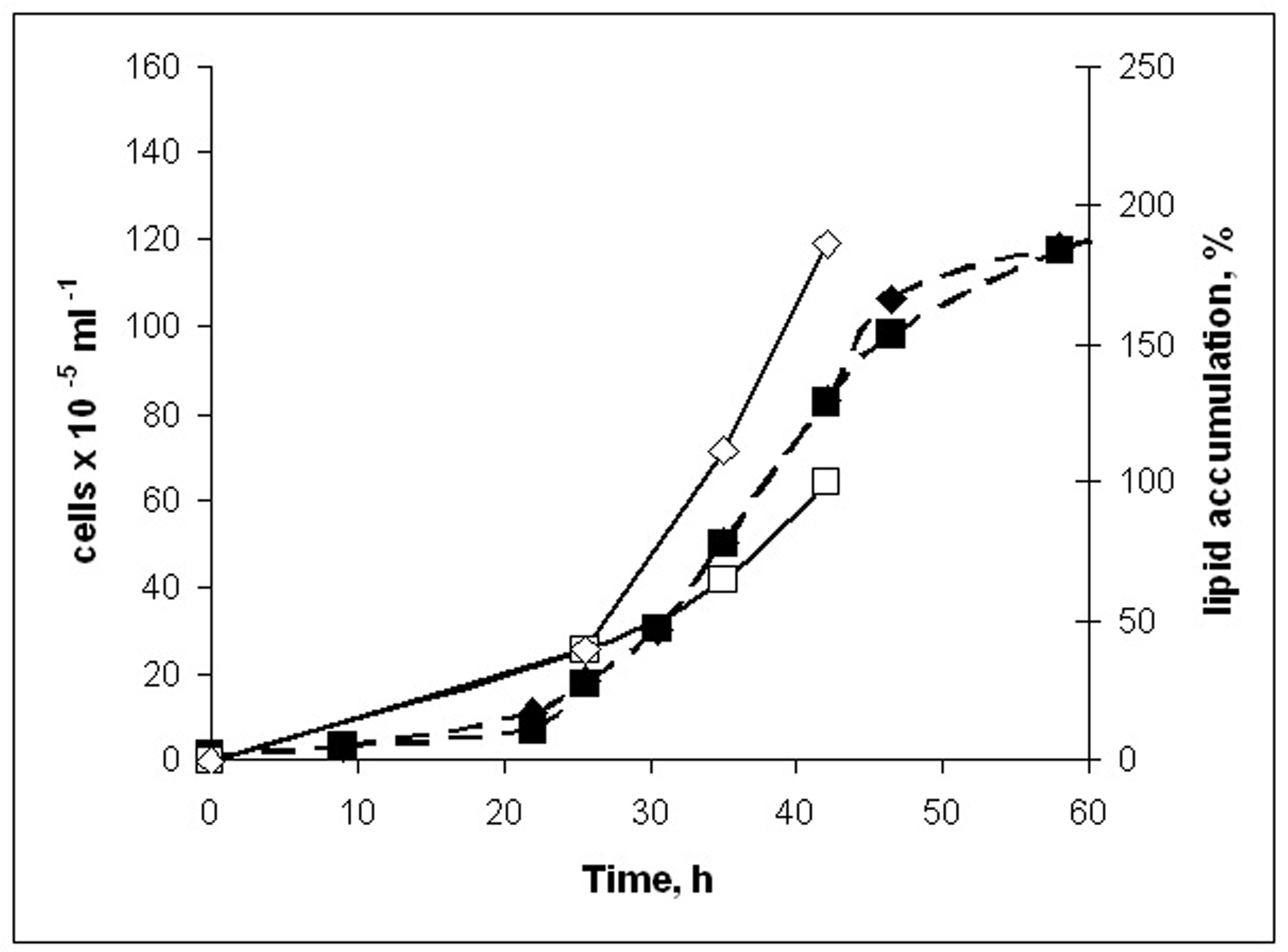


Reference:

1. Kimura K, Yamaoka M, Kamisaka Y**: Rapid estimation of lipids in oleaginous fungi and yeasts using Nile red fluorescenc**e*. J Microbiol Method*s 2004**, 5**6:331-338.
