# Supplementary figures and images for: Growth of Chlamydomonas reinhardtii in acetate-free medium when co-cultured with alginate-encapsulated, acetate-producing strains of Synechococcus sp. PCC 7002
Source: Biotechnol Biofuels. 2014 Oct 18;7:154. doi: 10.1186/s13068-014-0154-2 (PMC4216383; doi:10.1186/s13068-014-0154-2)

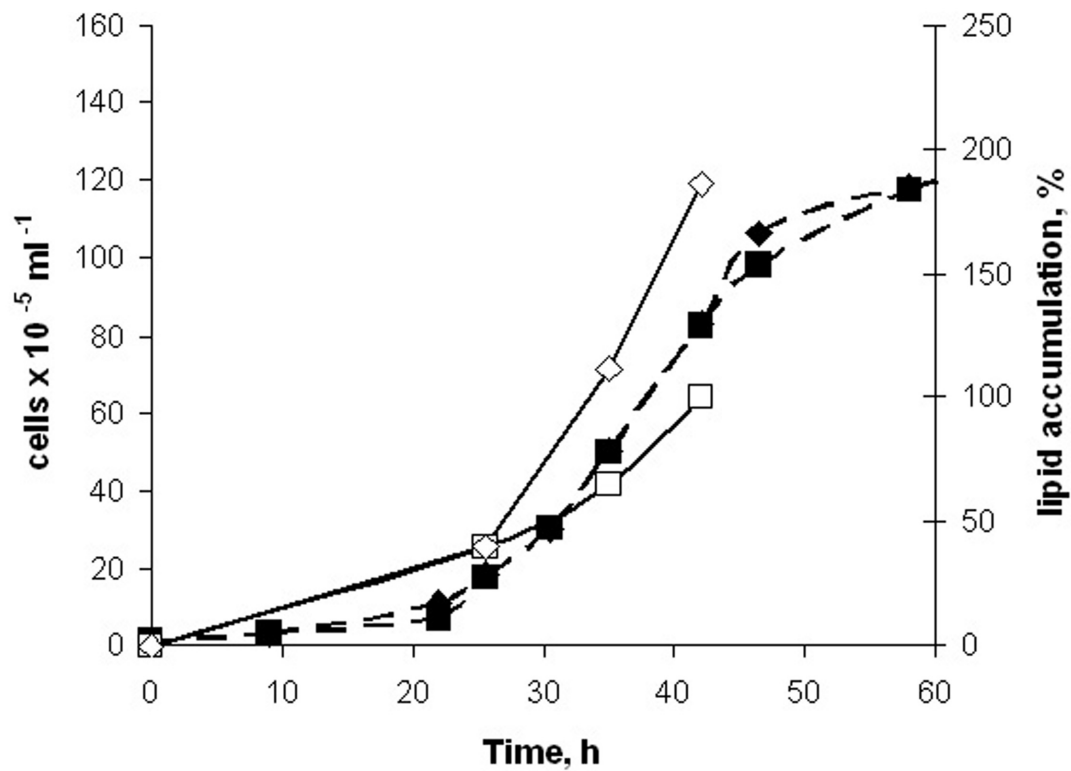

Supplement: Additional file 2: Figure S1. — Growth and lipid accumulation of wild-type and sta6 mutant C. reinhardtii. Cultures of wild-type (closed squares, dotted line) and sta6 mutant (closed diamonds, dotted line) C. reinhardtii were grown on modified TAP media at 30°C in the presence of acetate. The lipid accumulation in wild-type (open squares, solid line) and sta6 mutant (open diamonds, solid line) C. reinhardtii cells was determined with Nile red stain after a 10-min incubation [1]. The amount of lipids accumulated after 42 hours growth of wild-type C. reinhardtii cells was chosen as 100%. [file 13068_2014_154_MOESM2_ESM.pdf]
